# Circular RNAs as Potential Biomarkers in Breast Cancer

**DOI:** 10.3390/biomedicines10030725

**Published:** 2022-03-21

**Authors:** Fatima Domenica Elisa De Palma, Francesco Salvatore, Jonathan G. Pol, Guido Kroemer, Maria Chiara Maiuri

**Affiliations:** 1 Equipe 11 Labellisée Par La Ligue Nationale Contre Le Cancer, Centre de Recherche Des Cordeliers, Inserm U1138, Université de Paris Cité, Sorbonne Université, 75006 Paris, France; pol_jonathan@yahoo.fr (J.G.P.); kroemer@orange.fr (G.K.); 2 Metabolomics and Cell Biology Platforms, Gustave Roussy Cancer Campus, 94800 Villejuif, France; 3 Department of Molecular Medicine and Medical Biotechnology, University of Naples “Federico II”, 80131 Naples, Italy; salvator@unina.it; 4 CEINGE-Biotecnologie Avanzate, 80145 Naples, Italy; 5 Inter-University Center for multifactorial and multi genetic chronic human diseases, “Federico II”-Naples, Tor Vergata-Roma II, and Chieti-Pescara Universities, 80131 Naples, Italy; 6 Institut Universitaire de France, 75005 Paris, France; 7 Department of Biology, Institut du Cancer Paris CARPEM, Hôpital Européen Georges Pompidou, AP-HP, 75015 Paris, France; 8 Department of Pharmacy, University of Naples “Federico II”, 80131 Naples, Italy

**Keywords:** breast cancer, circular RNA, non-coding RNA, long non-coding RNAs, circulating RNA, circulating circRNAs, biomarker, diagnostic biomarker, microRNA, microRNA sponge

## Abstract

Due to the high heterogeneity and initially asymptomatic nature of breast cancer (BC), the management of this disease depends on imaging together with immunohistochemical and molecular evaluations. These tests allow early detection of BC and patient stratification as they guide clinicians in prognostication and treatment decision-making. Circular RNAs (circRNAs) represent a class of newly identified long non-coding RNAs. These molecules have been described as key regulators of breast carcinogenesis and progression. Moreover, circRNAs play a role in drug resistance and are associated with clinicopathological features in BC. Accumulating evidence reveals a clinical interest in deregulated circRNAs as diagnostic, prognostic and predictive biomarkers. Furthermore, due to their covalently closed structure, circRNAs are highly stable and easily detectable in body fluids, making them ideal candidates for use as non-invasive biomarkers. Herein, we provide an overview of the biogenesis and pleiotropic functions of circRNAs, and report on their clinical relevance in BC.

## 1. Introduction

Breast cancer (BC) is a non-lethal malignancy when diagnosed at an early stage [1]. In the last decade, the 5-year survival rate of BC patients has increased, with deaths due to BC dropping by more than 20% [2,3,4]. These encouraging observations result from improved therapeutic strategies as well as from earlier detection, the latter resulting from the widespread use of screening mammography in women of >40 years of age [4]. These clinical advances were also achieved thanks to molecular profiling that provides crucial information on one of the main scourges of BC, namely tissue heterogeneity [1,5].

Depending on the expression profile of key histological markers (i.e., oestrogen receptor (ER), progesterone receptor (PR), human epidermal growth factor receptor 2 (HER2) and Ki67), BC can be classified into four main subtypes: luminal A (lumA), luminal B (lumB), triple-negative (TNBC) and HER2-related BCs [1,6]. The heterogeneity of BC complicates its diagnostic and treatment decision-making, as well as prediction of the therapeutic response and survival [5,7]. Among all BC subtypes, lumA BC shows a better prognosis due to a high expression of druggable ER and PR [7,8]. By contrast, the TNBC subtype does not express ER, PR and HER2, leading to resistance to hormone and targeted treatments and unfavorable clinical outcome [1,6,7]. Therefore, determining the status of the diagnostic and predictive indicators is essential for the management of BC patients.

In addition to the established histological classification, other candidate biomarkers are being investigated. These include genetic features (mutations affecting one or multiple genes), epigenetic alterations (e.g., DNA methylation, non-coding RNAs [ncRNAs]), as well as protein analytes [8,9,10,11,12]. Detection of pathogenic mutations in the tumor suppressor genes *BRCA1* and *BRCA2* has greatly improved the management of BC patients and their relatives [13]. Indeed, *BRCA* testing predicts not only an increased risk of developing BC but also the responsiveness to platinum-based chemotherapy and to inhibitors of poly(ADP-ribose) polymerase (PARP) [14,15,16,17,18,19]. Nowadays, several multigene signature assays, including MammaPrint, Oncotype Dx and Prosigna/Prediction Analysis of Microarray50 (PAM50), have been approved for the diagnosis of early-stage BC, classification of molecular BC subgroups, and prediction of tumor recurrence [8,12]. Moreover, although small (miRNA) and long (lncRNA) non-coding RNAs have not (yet) been translated from bench to clinic in BC, accumulating evidence suggests their clinical utility as biomarkers in BC [9,11]. In the context of BC heterogeneity, profiling certain RNAs may complement conventional diagnostic methods. For example, in a recently published study, we have demonstrated that the abundance of a novel lncRNA, LINC01087, can discriminate the luminal and TNBC BC subtypes with high specificity [20]. 

Current methods applied for the diagnosis and prognosis of BC are invasive, expensive and time-consuming. Therefore, efforts are being done to characterize easily detectable biomarkers to ease the clinical management of BC patients. In this scenario, circular RNAs (circRNAs) are emerging as an attractive new class of highly stable and non-invasive diagnostic, prognostic and predictive biomarkers [21,22]. CircRNAs are a unique type of ncRNAs discovered in the 1970s and characterized by a loop structure [23]. Although they have been originally thought as junk biomolecules derived from splicing errors, circRNAs have demonstrated significant involvement in a variety of human diseases, such as diabetes mellitus, cardiovascular and neurological diseases, as well as in tumorigenesis, metastasis, cancer recurrence and multidrug resistance in neoplastic diseases [22,24,25].

This review article describes the current knowledge about the application of circRNAs as biomarkers in BC. We will first summarize circRNA biogenesis and their mode of action. Subsequently, we will discuss the clinical potential of circRNAs as tissue and circulating biomarkers for early detection, diagnosis and prognostication of breast cancer. Finally, we outline recent progress about circRNAs as useful predictors of resistance to therapeutic agents.

## 2. Biogenesis and Functions of Circular RNAs

**Figure 1:**
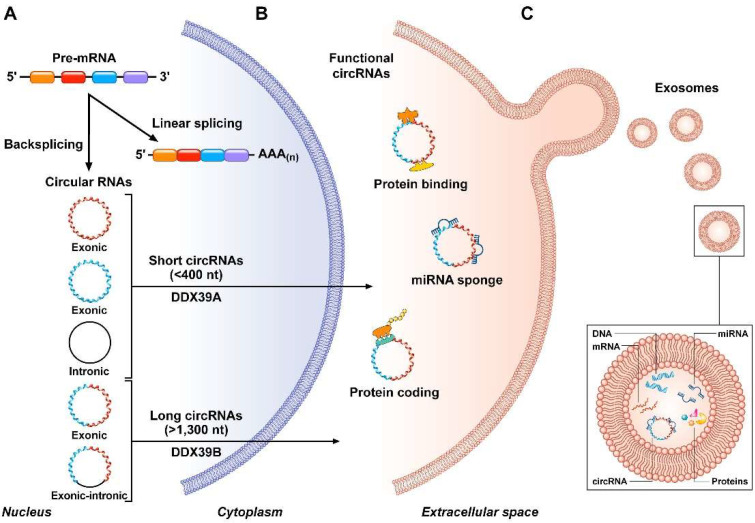
CircRNA biogenesis and functions. (**A**) Circularization of circRNAs, including exonic, intronic, and exonic-intronic circRNAs, arises from pre-mRNA transcripts through different mechanisms of back-splicing. After formation, circRNAs are transported from the nucleus to the cytoplasm by RNA helicases (DDX39A, or DDX39B) in a size-dependent manner. (**B**) CircRNAs have different mechanisms of action. Indeed, they can interact with proteins, encode for proteins, and function as microRNA sponge (mainly). (**C**) CircRNAs can be further released into body fluids as cell-free circRNAs or enriched in extracellular vesicles (e.g., exosomes).

Circular RNAs are a new type of evolutionary conserved single-stranded long ncRNAs that are characterized by a covalently closed loop without cap and polyadenilated (polyA) tail [25,26,27] (Figure 1). They are generated in the nucleus from precursor messenger RNAs (pre-mRNA) through different mechanisms, involving: (i) the exon skipping machinery, also known as “lariat-driven circularization”; and/or (ii) non-canonical mechanism of splicing (mostly), called back-splicing (or “intron-pairing”) wherein the 5′ end of a pre-mRNA upstream exon is spliced with the 3′ end of a downstream exon [24,26,28]. These processes of synthesis can be mediated and regulated by several factors comprising flanking inverted repeats and RNA binding proteins (RBPs) (e.g., quaking [QKI], muscleblind-like protein [MBL], RNA-specific adenosine deaminase enzyme [ADAR]) [24,29,30]. Notably, QKI and MBL proteins promote circRNA biogenesis, whereas ADARs inhibit it [30]. Additionally, epigenetic changes may control circRNA production [27,29,31]. Once generated, most circRNAs can be exported (through under investigated processes involving ATP-dependent RNA helicases) from the nucleus to the cytoplasm where they resist exonuclease-mediated digestion (mainly RNase R) and accumulate [26,29] (Figure 1). Due to their ring structure, circRNAs are remarkably stable molecules. Their half-life exceeds 48 h, which is twice as long than their linear counterparts [29].

Depending on the structural characteristics and on the mechanism of circularization, circRNAs are currently classified into three main types, namely circular intronic (ciRNA), exonic circRNAs (EcircRNA), and exon-intron circRNAs (EIciRNA) (Figure 1) [26,31]. 

EcircRNAs, from which the introns are spliced out, are mostly distributed in the cytoplasm and represent about 80% of total circRNAs [32]. Their circularization occurs via either exon skipping or back-splicing and they can contain one single or multiple exons [26]. CiRNAs consist only of introns and are mainly localized in the nucleus. They originate during splicing from intron lariats that escaped the debranching enzyme [32]. EIciRNAs, which are characterized by sequences derived from both exons and introns, are abundant in the nucleus and undergo similar biogenesis to eciRNAs (Figure 1) [33].

In contrast to their genesis, the degradation of circRNAs is understudied to date and seems to involve both exo- and endo-nucleases [29,34].

CircRNAs play crucial biological roles at the transcriptional and post-transcriptional levels. For instance, they can interact with RNA-binding proteins to act as protein scaffolds and protein sponges, or recruit proteins to specific cellular locations [26,35] (Figure 1). In addition to forming RNA-protein complexes, circRNAs can alter DNA methylation in the promoter of their parental gene to regulate their expression and functions [26,29,36]. Moreover, despite lacking 5′-cap and 3′-poly(A) tail, recent studies suggest that circRNAs may have a protein-coding potential [37,38,39,40] (Figure 1). For instance, circ-ZNF609 can be translated into a protein in a cap-independent, but splicing-dependent, manner [38]. Recent evidence revealed the potential of certain circRNAs like circRFWD2 to form pseudogenes [41,42]. Nevertheless, the main role of circRNAs seems to act as microRNA (miRNA) sponges [43] (Figure 1). In other studies, they appeared to function as competitive endogenous RNAs (ceRNAs) as they can bind complementary target regions in miRNAs, thus regulating gene expression and consequently downstream signaling pathways through the inhibition of miRNA activity. These pleiotropic properties explain the intricate involvement of circRNAs in tumorigenesis [25,31,44]. 

## 3. Circular RNAs as Tissue Biomarkers in Breast Cancer

CircRNAs are evolutionarily conserved ncRNAs that demonstrate tissue-specific expression. Due to their deregulated expression, the detection of certain circRNAs may implement the diagnosis and prognosis of BC patients as well as the prediction of response to therapy [21,45,46]. Substantial attention has been devoted to the quantification of single circRNA or signatures of circRNAs in tumor biopsies and cell lines. RNA-sequencing (RNA-seq) and/or microarray techniques are commonly used for the detection of circRNAs, while RT-qPCR or digital droplet PCR are secondarily applied for validating the results [47]. In this setting, increased expression of circRNAs has been associated with breast tumor status [47]. Moreover, different computational approaches dedicated to the study of circRNAs are emerging [48,49,50]. Collectively, these studies highlighted the potential of circRNAs as clinically relevant biomarkers in BC tissues as summarized in Table 1. Of note, the molecular and cellular mechanisms linking circRNAs deregulation and tumor progression remain poorly understood due to limited preclinical research on this topic. 

### 3.1. Oncogenic and Tumor-Suppressive Circular RNAs as Biomarkers of Breast Carcinogenesis

In 2016, Zheng and collaborators identified circHIPK3 as abundantly expressed in seven tumor tissues with respect to six normal tissues using an RNA-seq approach [54]. Accordingly, other studies confirmed the upregulation of circHIPK3 in tumor tissues and cell lines as compared to normal samples [55,56].

In BC, a study conducted by Lu et al. provided a profile of deregulated circRNAs (hsa_circ_103110, hsa_circ_104689, hsa_circ_104821) with diagnostic potential in infiltrating ductal carcinoma [64]. A previous study had also evoked the predictive value of the circRNA profile in in situ and invasive ductal carcinoma [87]. Moreover, the circRNAs hsa_circ_0006743 and hsa_circ_0002496 were identified as upregulated in the early stage of BC, suggesting their interest in the early diagnosis of the disease [62]. Accordingly, the aberrant expression of hsa_circ_0005046 and hsa_circ_0001791 showed remarkable value for the early detection of BC (AUC = 0.77 and 1, respectively) and for predicting the clinical outcome [61]. A high level of additional circRNAs (e.g., circHMCU, hsa_circ_0055478 [circPTCD3], hsa_circ_0005728 [circUBE2D2], hsa_circ_0087784 [circRNF20], hsa_circ_008717 [circABCB10], hsa_circ0005230, circRPPH1_015) also demonstrated significance for the diagnosis and dismal prognosis of BC [51,57,58,59,60,63,69,79]. Other circRNAs have been associated with clinicopathological features. For instance, elevated expression of circDENND4C was associated with advanced breast tumor stages (lymph node engagement, metastasis) [52,53]. Another example concerns hsa_circ_0103552 whose overexpression in BC patients correlated with clinical severity as well as dismal prognosis [65]. Thus, multiple circRNAs seem to harbor an oncogenic activity in breast tissue.

In parallel, some circRNAs show tumor suppressor activity. For instance, circCCDC85A and hsa_circ_0072309 are found down-expressed in BC tissues and/or cell lines when compared to normal controls [66,69]. Similarly, circVRK1 is downregulated in BC [68]. Importantly, its high abundance has been associated with reduced tumor size, TNM stage, as well as better survival. In vitro, its expression inhibits proliferation and induces apoptosis in BC cells [68]. As another example, elevated expression of circLARP4 appeared as a biomarker of favorable prognosis in BC [67].

### 3.2. Circular RNAs as Biomarkers of Specific Breast Cancer Subtypes

To date, there remains a knowledge gap regarding the profile of circRNA across BC subtypes. CircRNAs have been particularly studied in TNBC which exhibits the most aggressiveness and resistance to chemotherapy [88]. In the latter subtype, circFBXW7 dysregulation coincided with a good prognosis [72]. Its level of expression negatively correlated with tumor size and lymph node metastasis. Overexpression of circFBXW7 inhibited murine TNBC cell proliferation in vitro and metastasis in vivo [72]. Conversely, a high expression of circEPSTI1, circAGFG1, and circKIF4A correlated with a dismal prognosis in TNBC [70,71,75]. In the same BC subtype, circUBAP2 expression was associated with advanced clinicopathological characteristics and poor overall survival [78]. Similarly, increased abundance of hsa_circ_0005320 (circSEPT9) was associated with advanced clinical stage and poor prognosis [77]. Moreover, in vitro and in vivo experiments corroborated its oncogenic role in breast tumorigenesis. In a recently published study, aberrant expression of circPDCD11 (hsa_circ_0019853) correlated with unfavorable survival and acted as an independent risk factor for TNBC prognosis [76]. Furthermore, circPDCD11 promoted TNBC progression by enhancing aerobic glycolysis [76]. CircGFRA1 has been described as upregulated in TNBC cell lines and tissues [73]. Kaplan-Meier curve analysis indicated that an elevated expression level of circGFRA1 predicted a poor outcome in BC. Additionally, circGFRA1 demonstrated interest as a therapeutic target [74]. Notably, the knockdown of circGFRA1 decreased the resistance of TNBC cells to paclitaxel by regulating the miR-361-5p/TLR4 pathway in the MDA-MB-231 cell line [74].

CircRNAs have not been particularly explored in luminal and HER2-related BC subtypes. In a research article published in 2016, Nair and colleagues attempted to determine signatures of circRNAs in distinct BC types using a bioinformatics approach called “Circ-Seq”. The latter is applied to transcriptomic data from BC clinical specimens extracted from the TCGA database [89]. In a microarray expression profile of circRNAs performed on BC cells, circASS1 was down-expressed in the MDA-MB-231 (TNBC) cell line but overexpressed in MCF7 (luminal) cells. Functional investigations corroborated the role of circASS1 in breast tumorigenesis [83]. This research requires further validation in other BC subtypes and tissue samples. Recently, differential expression of circRNAs between BC tissue subtypes has been inspected through a high-throughput microarray [80]. In particular, 140 upregulated and 95 downregulated circRNAs were identified. Among these, circTADA2A-E6 (hsa_circ_0006220) and circTADA2A-E5/E6 (hsa_circ_0043278) were significantly and uniformly downregulated across BC subtypes [80]. Conversely, low levels of hsa_circ_0044234 distinguished between TNBC and other BC subtypes, and were associated with high levels of Ki67, histological grade, and predicted worse clinical outcomes [85]. Thus, hsa_circ_0044234 demonstrated clinical usefulness as a discriminative diagnostic biomarker in TNBC (AUC = 0.82; *p* ≤  0.0001; 72.5% sensitivity and 83.64% specificity) [85]. Moreover, a recent study by Fan and collaborators evidenced a lower level of circNR3C2 (hsa_circ_0071127) in TNBC as compared to luminal BC tissues and cell lines [84]. Yet, overall high expression of circNR3C2 correlated with dismal prognosis [84]. Analogously, hsa_circ_001783 was more expressed in TNBCs than in the luminal and HER2^+^ BC subtypes [82]. Using BC cell lines, Tarrero and collaborators defined a set of circRNAs (circ_PGR_2-7, circ_CDH1_9-10, circ_ESR1_3-4, circ_NCOA3_4-9, circ_IGF1R_2, circ_GFRA1_5-7, circ_CDYL-4, circ_HIPK3_2, circ_RELL1_4-6, and circ_MAN1A_2-5) able to differentiate between samples of the luminal subtype and the others [90]. Another work evaluated the expression of circRNAs in 20 BC cell lines characterized by a luminal phenotype or TNBC epithelial or mesenchymal morphology [86]. The level of circSLC8A1 appeared significantly lower in the luminal than in the TNBC cell lines [86]. This result was confirmed in another study [81]. Furthermore, hsa_circ_0020397, also known as circDOCK1-1, showed high levels in luminal and epithelial TNBC cells, and a weak expression in mesenchymal TNBC cell lines [86].

Thus, circRNAs show a clinically relevant interest in the diagnosis of BC, in particular for its early detection and stratification into subtypes, as well as its prognosis.

## 4. Circular RNAs as Blood-Based Biomarkers in BC

In the diagnostic workflow of BC, imaging examinations must be integrated with cytological and histological diagnoses, which require invasive procedures for tissue sampling. This procedure particularly improves the differential discrimination of BC subtypes.

Liquid biopsy is a minimally invasive procedure that limits the need for solid tissues and therefore can overcome complications of surgical biopsies [91,92]. This rapid and powerful method aims at diagnosing tumors by detecting and quantifying related biomarkers that circulate in biofluids (e.g., blood, serum, urine, gastric juice, breast milk) [92]. Due to their circularized and covalently closed structure, circRNAs are highly stable in the bloodstream [93], increasing their potential relevance as circulating biomarkers in BC [94] (Table 2).

Over the past years, cumulated investigations proved the presence of circRNAs in the “tumor circulome” [106]. Like other types of ncRNAs, circRNAs can travel in the peripheral blood as cell-free RNAs or within extracellular vesicles (exosomes) [107,108,109]. A majority of studies revealed enrichment of circRNAs in serum or plasma samples of BC patients with respect to healthy samples [99,100,101,102,103,104,108,110,111]. Functional wet-lab experiments are anticipated to determine the involvement of circulating circRNAs in breast carcinogenesis.

### 4.1. Plasma Circular RNAs as Biomarkers in BC

The expression of four circRNAs, namely hsa_circ_0069094, hsa_circ_0079876, hsa_circ_0017650, and hsa_circ_0017536, was measured in plasma specimens of BC patients [102]. Both hsa_circ_0017650 and hsa_circ_0017536 were significantly upregulated in the bloodstream but not in breast tissue of the cohort as compared to healthy donors. By contrast, hsa_circ_0069094 and hsa_circ_0079876 showed the same altered abundance in both plasma and tissue specimens [102].

Hsa_circ_0001785 (circELP3) is considered a promising biomarker for early detection of BC [99]. By using a microarray approach, Yin and collaborators revealed a significant overexpression of hsa_circ_0001785 in plasma samples of BC patients (n = 5) as compared to healthy samples (n = 5). Its deregulated expression and diagnosis value were validated in a larger cohort (n = 40) by RT-qPCR and ROC curve analysis (AUC = 0.789), respectively. Changes in the level of expression of hsa_circ_0001785 were associated with worse tumor grade and TNM status. Remarkably, the abundance of hsa_circ_0001785 significantly decreased in post-operative plasma samples of BC patients when compared to pre-operative specimens. Concomitantly, a lower level of another circRNA, circFAF1, was witnessed in serum samples of BC patients prior to medical interventions (surgery and/or chemotherapy) as compared to post-treatment specimens [110]. Similarly, post-operative plasma samples from BC patients presented low levels of cell-free hsa_circ_0008673 with respect to pre-operative specimens [100]. Of note, further investigations will require a post-treatment follow-up of the circulating hsa_circ_0001785 and hsa_circ_0008673 to determine whether their detection could predict tumor recurrence. The measurement of hsa_circ_0008673 in plasma was distinguishing between BC patients and healthy controls. Moreover, increased detection level of circulating hsa_circ_0008673 was positively associated with clinicopathological features (e.g., larger tumor size, distant metastasis, ER and PR positive status) and predicted unfavorable overall and disease-specific survival. Mechanistically, the knockdown of hsa_circ_0008673 in BC cell lines impaired their proliferation, viability, as well as migration and invasion abilities. Thus, hsa_circ_0008673 displays a pro-oncogenic activity in breast tissue [100]. Furthermore, Zhang and collaborators recently reported that hsa_circ_0008673 regulates breast cell malignancy by upregulating CFL2 via the sponging of miR-153-3p [101].

An increased expression of hsa_circ_0104824 was observed in the plasma of 83 BC patients. It correlated with tumor stage and grading and exhibited a diagnostic value mainly in subjects with ER and PR positive BC [103]. Moreover, bioinformatics analyses revealed the role of hsa_circ_0104824 as a miRNA sponge, as well as its enrichment in oncological processes, such as proliferation and metastasis [103]. In a pilot study, Smid et al. measured by RT-qPCR circCNOT2 (hsa_circ_0008285) in plasma samples of four BC patients. Data supported interest in circCNOT2 as a non-invasive biomarker to predict response to chemotherapy [98]. CircBCBM1 (hsa_circ_0001944) was also detected in the plasma of BC patients. Interestingly, upregulation of this circRNA was observed in participants who developed brain metastasis as compared to non-metastatic BCs. Thus, CircBCBM1 measurement would benefit the diagnosis and prognosis of BC, and might constitute a novel target for the management of BC brain metastases [111].

### 4.2. Serum Circular RNAs as Biomarkers in BC

In a recent investigation, an upregulation of hsa_circ_0007255 (circKIF4A) was observed by RT-qPCR in serum samples of BC subjects (n = 50) versus healthy controls (n = 50) [105]. On top of this diagnostic potential, detection of a high level of hsa_circ_0007255 was also indicative of a poor prognosis in TNBC tissues. Experimentally, a dysregulation of hsa_circ_0007255 played an oncogenic role in breast tissue by modulating the miR-335-5p/SIX2 axis [105].

The abundance of circCDYL was measured in the serum of early (n = 30), benign (n = 14) and metastatic (pulmonary and hepatic distant sites; n = 18) BC patients using droplet digital PCR [104]. CircCDYL detection level segregated patients according to the severity of their pathology. More precisely, weak amounts of circulating circCDYL were quantitated in benign samples, as opposed to intermediate and strong levels in early and metastatic BCs, respectively. Intriguingly, real-time kinetics of serum circCDYL during chemotherapy revealed that the level of this circulating circRNA correlated with the response to therapy in metastatic BC patients. Along this line, high levels of serum circCDYL were associated with a worse prognosis in these patients [104].

### 4.3. Exosomal Circular RNAs as Biomarkers in BC

Exosomes are extracellular vesicles (EVs) whose content reflects some characteristics of their parental cell. CircRNAs can be enriched in exosomes released in the bloodstream [107] (Figure 1). Recently, Lin et al. described a signature of nine circRNAs isolated from plasma EVs (hsa_circ_0002190, hsa_circ_0007177, hsa_circ_0000642, hsa_circ_0001439, hsa_circ_0001417, hsa_circ_0005552, hsa_circ_0001073, hsa_circ_0000267, hsa_circ_0006404) and collectively referred to as BC_ExoC_. Upregulation of BC_ExoC_-related circRNAs appeared as a non-invasive determinant of early BC [97]. Similarly, the profile of circRNA in serum exosomes of BC patients versus healthy donors has been compared by Yang and collaborators using next-generation sequencing, subsequent validation by RT-qPCR, and bioinformatics analyses [96]. Among the deregulated circRNAs identified, hsa_circRNA_0005795 and hsa_circRNA_0088088 emerged as the most significantly downregulated and upregulated in BC with respect to normal samples, respectively. Likewise, another study reported the significant overexpression of five circRNAs (i.e., hsa_circ_0009634, hsa_circ_0020707, hsa_circ_0064923, hsa_circ_0104852, and hsa_circ0087064) in exosomes derived from BC cell lines, as well as from metastatic BC patients using transcriptomics approaches [95].

Altogether, circRNAs in liquid biopsy are promising biomarkers in human BC. Among the circulating circRNAs that have been identified, the majority was described for their diagnostic utility, but only a few showed a strong clinical potential, as demonstrated by low AUC values. Further investigations, including basics and translational research, are mandatory in order to potentiate and validate such potential.

## 5. Circular RNAs as Biomarkers of Resistance to BC Treatments

Surgery, standard and targeted chemotherapy, radiotherapy, hormone therapy and immunotherapy compose the current armamentarium against BC [6,7,26]. For each patient, establishing the therapeutic strategy requires a multidisciplinary team of clinicians to envision a variety of clinicopathological parameters (e.g., age, general health, body mass index, menopausal status, hormone receptor, HER2, nodal status, stage of the tumor) as well as genetic and molecular tumor determinants (e.g., mutations, gene expression profile) [6].

Patients with hormone receptor-positive tumors (luminal A and HER2-negative luminal B subtypes) are administered endocrine therapy (e.g., tamoxifen, fulvestran, aromatase inhibitors), which can be combined with standard chemotherapy and cyclin-dependent kinase 4/6 inhibitors (i.e., palbociclib, abemaciclib, ribociclib) [112]. HER2-positive luminal B and HER2-positive tumors benefit from chemotherapy in combination with monoclonal antibodies (e.g., trastuzumab, pertuzumab), and/or inhibitors of the tyrosine kinase domain of HER2 (e.g., lapatinib), together with endocrine therapy [113,114,115]. Regarding the aggressive TNBC subtype, chemotherapy is the reference treatment [116]. Chemotherapeutic regimens comprise some compounds that target the DNA repair complex (platinum drugs, taxanes), TP53 (taxanes) or cell proliferation (anthracycline-containing regimen) [117]. Yet, comprehensive molecular and functional studies have identified effective strategies that can improve the outcome and response to treatment of TNBC patients. These novel treatments include immune checkpoint inhibitors (anti-PD-1/PD-L1 antibodies), antibody-drug conjugates (ADCs), or again PARP inhibitors among others [116,117,118].

Nevertheless, resistance to treatments remains one of the main challenges in BC and an obstacle in increasing the survival rate of patients [119,120,121]. In this scenario, personalized medicine may significantly improve patient outcomes. To reach this goal, circRNAs appear as promising predictive biomarkers as accumulating evidence suggests their ability to regulate BC cell sensibility to therapies [45,122] (Table 3).

### 5.1. Circular RNAs as Biomarkers of Resistance to Hormone Therapy

Circ_0025202 has been reported as a potential predictive biomarker of BC resistance to tamoxifen (Table 3), one of the oldest and most successful endocrine therapies for ER-positive BCs [130]. In detail, circRNA_0025202 appeared downregulated in TAM-resistant MCF7 cells using an RNA-seq approach. Moreover, circRNA_0025202 overexpression reverted the sensitivity to tamoxifen in BC cells and mouse experimentations. Conversely, another study demonstrated the potential of hsa_circ_0003218, also known as circBMPR2, to reduce tamoxifen resistance through mir-553/USP4 axis [129] (Table 3).

### 5.2. Circular RNAs as Biomarkers of Resistance to Chemotherapy

It has been demonstrated that the inhibition of hsa_circ_0001946 (cirCDR1as) increases the sensitivity to 5-fluorouracil and cisplatin of originally resistant BC cells [123,124]. Xu and collaborators also provided information on the role of circSMARCA5 (hsa_circ_0001445) in affecting the sensitivity of BC to cisplatin [125]. CircSMARCA5 is present at a lower level in tissue and plasma samples of BC patients than in normal specimens. Interestingly, the study evidenced that restoration of circSMARCA5 levels improved the chemosensitivity of BC cells and tumors [125] (Table 3).

Several lines of evidence also revealed the inhibitory role of many circRNAs (e.g., circKDM4C, circUBE2D2, hsa_circ_0092276, circLARP4) in resistance to doxorubicin [67,79,126,128] (Table 3). Notably, circKDM4C (hsa_circ_0001839) downregulation has been associated with poor prognosis and lymphatic metastasis in BC tissues [126]. Moreover, doxorubicin-resistant cells showed a lower level of circKDM4C as compared with sensitive parental cells. Mechanistically, the knockdown of circKDM4C increased the resistance to doxorubicin. By contrast, its overexpression increased the sensitivity to doxorubicin in vitro and in vivo. Thereby, circKDM4C may be a potential biomarker to predict the response to doxorubicin in BC patients [126]. Similarly, another recently published study demonstrated the involvement of circUBE2D2 (hsa_circ_0005728) in cancer progression and doxorubicin resistance in TNBC, by acting at the cellular level as a sponge of miR-512-3p, in turn resulting in the upregulation of CDCA3 expression [79].

A signature of 18 differentially expressed circRNAs has been identified by comparing doxorubicin (also known as adriamycin or ADM)-resistant MCF7 BC cells versus parental MCF7 cells, using a high-throughput circRNA microarray approach [127]. Among them, the expression of hsa_circ_0006528 was significantly associated with ADM-resistant BC cells and tissues (Table 3). Remarkably, the sensitivity of ADM-resistant cells to ADM was increased after forced downregulation of hsa_circ_0006528 using RNA interference method.

Data from different investigations also highlighted the involvement of circRNAs in the resistance to taxanes. For example, circABCB1, circEPHA3.1 and circEPHA3.2 contribute to the resistance to docetaxel via the PI3K-Akt and AGE-RAGE signaling pathways [131] (Table 3). CircAMOTL1 has been reported to inhibit cell apoptosis when exposed to paclitaxel (PTX) in vitro [132] (Table 3). Accordingly, circRNF111 (hsa_circ_0001982) promoted resistance to PTX in BC cells in vitro and enhanced PTX sensitivity in vivo [133] (Table 3). Similarly, CircABCB10 interferes with PTX responsiveness [134] (Table 3). Its expression was higher in PXT-resistant BC cells and tissues than in corresponding parental samples. The silencing of circABCB10 decreased the proliferation of BC cells through the upregulation of the phosphatase DUSP7 via the sponging of the miRNA let-7a-5p. In vivo investigation raised concordant results, therefore comforting a contribution of circABCB10 to PTX resistance [134].

### 5.3. Circular RNAs as Biomarkers of Resistance to Radiotherapy and Immunotherapy

In a recently published paper, Zhao and colleagues demonstrated that upregulation of circABCB10 increased BC resistance to radiotherapy via the miR-223-3p/PFN axis [135] (Table 3).

Li and colleagues reported high levels of circHER2 in TNBC [136]. In comparison to circHER2-negative TNBCs, patients bearing circHER2-positive TNBC tumors presented an unfavorable outcome. Notably, this work reported that circHER2 encodes for a 103-amino acid protein named HER2-103. This protein seemed to participate in the proliferation and invasion of TNBC cells. Interestingly, HER2-103 shared some sequences with the CR1 domain of HER2 thus sensitizing it to antagonization by pertuzumab. Along this line, pertuzumab reduced the progression of circHER2 and HER2–103 positive TNBC tumors [136]. Interestingly, although pertuzumab is commonly used for HER2 blockade in HER2-positive BC patients only, this study showed that TNBC patients that express circ-HER2/HER2-103 could benefit from pertuzumab as well. 

In summary, circRNAs may serve as predictors for responsiveness to chemo-, radio-, immuno- and hormone-therapies. Further clinical investigations are encouraged for validation.

## 6. Discussion

Over the last decade, scientific knowledge on circRNAs in BC has considerably expanded. CircRNAs gradually revealed multiple functions as oncogenic or tumor suppressor molecules in different aspects of breast carcinogenesis (e.g., angiogenesis, cell proliferation, apoptosis, epithelial to mesenchymal transition, metastasis, drug resistance) [26]. They also play important physiological roles such as cellular differentiation and maintenance of stem cell pluripotency [137]. However, only a small subset of the circRNAs identified (mainly by RNA-seq technique) has been deeply studied or validated by independent methods. Being deregulated in BC, the assessment of individual circRNA or signature of circRNAs expression may have clinical relevance for patient management [94]. Numerous circRNAs have been depicted as potential biomarkers for BC diagnosis (mainly) and prognostication. In particular, their assessment can facilitate early detection of BC which is critical to avoid morbidity and metastasis, and thus extend survival [45,138]. Among them, ten circRNAs (i.e., hsa_circ_0005046, hsa_circ_0001791, hsa_circ_006054, hsa_circ_100219, circVRK1, circAGFG1, circSEPT9, circTADA2A-E6, circTADA2A-E5/E6, and hsa_circ_0044234) showed a high potential as diagnostic biomarkers (AUC > 0.7).

Their role in predicting responsiveness to radiotherapy and to various therapeutic compounds has also been described [122,127]. In particular, altered abundance of circRNAs was able to increase the chemosensitivity to doxorubicin (circKDM4C, circUBE2D2, and circLARP4), pertuzumab (circHER2), cisplatin (circSMARCA5), tamoxifen (circRNA_0025202, circBMPR2), 5- fluorouracil (cirCDR1as) and taxanes (circABCB1, circEPHA3.1, circEPHA3.2, circABCB10, and circRNF111) [79,123,129,130,131,133,134,135,136]. Furthermore, some evidence encourages the application of circRNAs as therapeutic agents thanks to their regulatory capacity [139].

Owing to their high stability in human body fluids (e.g., blood, urine, breast milk), circRNAs may have a pivotal role as biomarkers also in the blood circulation of BC patients [94,106,108]. They have been detected in whole blood, serum, plasma, as well as in isolated exosomes [95,97,107,108]. Inter alia, six circulating circRNAs (i.e., hsa_circ_0017650, hsa_circ_0001785, hsa_circ_0008673, hsa_circ_0104824, circKIF4A and BCexoc) demonstrated a strong diagnostic value (AUC >0.7). The BCexoc biomarker signature of nine circRNAs stands out for its potential clinical utility. Indeed, considering the successful clinical application of tissue-based multigene assays, such as MammaPrint and OncotypeDx, the quantitative measurement of BCexoc seems to be entirely feasible and hence might be introduced into the practical management of early BC.

Currently, the majority of the published studies have conducted their experimental research on cell lines and tissue specimens from small discovery cohorts, often without validation in independent cohorts. Moreover, differently from other ncRNAs, only a few studies showed the potential role of circRNAs to differentiate between BC subtypes, with a particular focus on TNBC [85,89]. Interestingly, hsa_circ_0044234 demonstrated its potential role in discriminating TNBC from non-TNBC patients (AUC = 0.82, *p* < 0.0001, sensitivity = 72.5%, specificity = 83.6%)**.** Despite the fact that luminal BC has a relatively good prognosis, a subset of patients do show a dismal outcome and do not respond to hormone therapy. Hence, there is a critical need to identify new biomarkers for early detection and more effective therapies also in luminal subtypes, as well as HER2-related tumors.

This area of research concerning the potential utility of circRNAs as tumor markers is as attractive as it is complex and challenging, especially for its clinical applicability. Significant insights in the field have proved their clinically valuable potential role for personalized diagnostic and treatment. However, as opposed to other ncRNAs, their clinical relevance as biomarkers in BC has not been assessed in large clinical cohorts. Currently, their potential value as diagnostic, prognostic, and therapeutic biomarkers is being evaluated as illustrated by the registration of ten recruiting clinical trials in a variety of human diseases (e.g., neuroendocrine tumors, ischemic stroke, pancreaticobiliary cancers, pancreas adenocarcinoma) (source: clinicaltrials.gov, accessed on 1 Febuary 2022).

We believe that future investigations should overcome the disturbing methodological heterogeneity and absence of methodological standardization. To progress to clinical translation, larger and better-characterized cohorts, proper validation techniques, appropriate control groups, together with more information on tissue heterogeneity of BC will be needed.

We envision that circRNAs will revolutionize and streamline the complicated diagnostic workflow of BC and will usher new therapeutic scenarios that will have full potential to improve the clinical outcomes of BC patients. Considering the power and versatility of circulating molecules, we would like to encourage scientific advances with respect to circRNA in the context of liquid biopsies.

## 7. Conclusions

CircRNAs have been recently discovered and their role in breast pathogenesis is recognized. In this Review, we have summarized unfolding research on these intriguing molecules in BC. Moreover, we have emphasized their potential clinical utility for the diagnostic and therapeutic management of BC.

Several lines of evidence demonstrate the likely utility of circRNAs as diagnostic, prognostic, and treatment-guiding biomarkers in BC. In particular circRNAs showed great potential in stratifying patients according to the BC subtype. The majority of the studies focused on the detection and quantification of circRNAs in BC tissue specimens from patients. In addition, recent research has focused on circulating circRNAs (found in the liquid phase of plasma or serum as well as bound to exosomes), considering the necessity of non-invasive and robust biomarkers.

Despite progress in the area, none of the proposed circRNAs has (yet) reached clinical approval as a biomarker. At present, knowledge gaps on circRNAs are exacerbated by the biological and the pre- and post-analytical variability, as well as by methodological challenges, impeding their clinical exploitation. It can be hoped that progressive methodological refinement and standardization will open the path to future prospective trials that definitively establish the clinical utility of circRNA measurements. 

## Figures and Tables

**Table 1:** List of circRNAs reported as potential biomarkers in breast cancer.

circRNA	circBase ID	Tissue Comparison	Expression in T	Clinical Interest	Diagnostic/Prognostic Value (ROC/KM Curve)	Experimental Approach
circABCB10	hsa_circ_008717	T vs. N	up	diagnosis	-	ex vivo, in vitro, in silico [51]
circDENND4C	-			diagnosis, prognosis	-	ex vivo, in vitro, in vivo, in silico [52,53]
circHIPK3	hsa_circ_0000284			diagnosis, prognosis	-	ex vivo, in vitro, in vivo, in silico [54,55,56]
circHMCU	hsa_circ_0000247			diagnosis, prognosis	HR = 3.09, *p* = 0.039	ex vivo, in vitro, in vivo, in silico [57]
circPTCD3	hsa_circ_0055478			diagnosis	-	ex vivo, in vitro, in vivo [58]
cirRNF20	hsa_circ_0087784			diagnosis, prognosis	-	ex vivo, in vitro, in vivo [59]
circRPPH1_015	hsa_circ_0000517			diagnosis, prognosis	-	ex vivo, in vitro, in vivo, in silico [60]
-	hsa_circ_0001791			early stage diagnosis	AUC = 1.0, *p* < 0.0001	ex vivo, in silico [61]
-	hsa_circ_0002496			early stage diagnosis	-	ex vivo, in silico [62]
-	hsa_circ_0005046			early stage diagnosis	AUC = 0.77, *p* = 0.02	ex vivo, in silico [61]
-	hsa_circ_0005230			diagnosis, prognosis	HR = 1.945, *p* = 0.042	ex vivo, in vitro, in silico [63]
-	hsa_circ_0006743			early stage diagnosis	-	ex vivo, in silico [62]
-	hsa_circ_103110			diagnosis	AUC = 0.63, *p* = 0.016	ex vivo, in silico [64]
-	hsa_circ_103552			diagnosis, prognosis	-	ex vivo, in vitro, in silico [65]
-	hsa_circ_104689			diagnosis	AUC = 0.61, *p* = 0.041	ex vivo, in silico [64]
-	hsa_circ_104821			diagnosis	AUC = 0.60, *p* = 0.031	ex vivo, in silico [64]
circCCDC85A			down	diagnosis	-	ex vivo, in vitro, in vivo, in silico [66]
circLARP4	-			diagnosis, prognosis	-	ex vivo, in vitro, in silico [67]
circVRK1	hsa_circ_0141206			diagnosis, prognosis	AUC = 0.720, Sensitivity = 61.7%, Specificity = 79.1%, HR = 0.375, *p* = 0.002	ex vivo, in vitro, in silico [68]
-	hsa_circ_006054			diagnosis	AUC = 0.71, *p* < 0.001	ex vivo, in silico [64]
-	hsa_circ_0072309			diagnosis, prognosis	-	ex vivo, in vitro, in vivo, in silico [69]
-	hsa_circ_100219			diagnosis	AUC = 0.78, *p* < 0.001	ex vivo, in silico [64]
-	hsa_circ_406697			diagnosis	AUC = 0.64, *p* < 0.008	ex vivo, in silico [64]
**circRNA**	**circBase ID**	**Tissue** **comparison**	**Expression in TNBC**	**Clinical interest**	**Diagnostic/prognostic value (ROC/KM curve)**	**Experimental approach**
circAGFG1	-	TNBC vs. N	up	diagnosis, prognosis	AUC = 0.767, HR = 6.072, *p* < 0.001	ex vivo, in vitro, in vivo, in silico [70]
circEPSTI1	-				-	ex vivo, in vitro, in vivo, in silico [71]
circFBXW7	hsa_circ_0001451				HR = 0.215, *p* = 0.001	ex vivo, in vitro, in vivo, in silico [72]
circGFRA1	hsa_circ_005239				-	ex vivo, in vitro, in vivo, in silico [73,74]
circKIF4A	-				-	ex vivo, in vitro, in vivo, in silico [75]
circPDCD11	hsa_circ_0019853				-	ex vivo, in vitro, in vivo, in silico [76]
circSEPT9	hsa_circ_0005320				AUC = 0.711, Specificity = 75%, Sensitivity = 63.3%, HR = 3.042, *p* = 0.012	ex vivo, in vitro, in vivo, in silico [77]
circUBAP2	hsa_circ_0001846				-	ex vivo, in vitro, in vivo, in silico [78]
circUBE2D2	hsa_circ_0005728				-	ex vivo, in vitro, in vivo, in silico [79]
circTADA2A-E6	hsa_circ_0006220		down		AUC = 0.855, *p* < 0.0001	ex vivo, in vitro, in vivo, in silico [80]
circTADA2A-E5/E6	hsa_circ_0043278				AUC = 0.94, *p* < 0.001	ex vivo, in vitro, in vivo, in silico [80]
**circRNA**	**circBase ID**	**Cell line** **comparison**	**Expression in TNBC**	**Clinical interest**	**Diagnostic/prognostic value (ROC/KM curve)**	**Experimental approach**
circSLC8A1	-	TNBC vs. luminal	up	diagnosis, BC subtypedistinction	-	ex vivo, in vitro, in vivo, in silico [81]
-	hsa_circ_001783	TNBC vs. luminal/HER2		diagnosis, prognosis	HR = 9.114, *p* = 0.001	ex vivo, in vitro, in silico [82]
circASS1	hsa_circ_0089105	TNBC vs. luminal	down	diagnosis	-	in vitro, in silico [83]
circNR3C2	hsa_circ_0071127	TNBC vs. luminal		diagnosis, prognosis	-	ex vivo, in vitro, in vivo, in silico [84]
-	hsa_circ_0044234	TNBC vs. non-TNBC		diagnosis, BC subtype distinction	AUC = 0.82, *p* < 0.0001, Sensitivity = 72.5%, Specificity = 83.6%, HR = 0.47, *p* = 0.058	ex vivo, in vitro, in silico [85]
**circRNA**	**circBase ID**	**Cell line** **comparison**	**Expression in luminal/epithelial TNBC**	**Clinical interest**	**Diagnostic/prognostic value (ROC/KM curve)**	**Experimental approach**
circDOCK1-1	hsa_circ_0020397	luminal/epithelial TNBC vs. mesenchymal TNBC cell lines	up	diagnosis, BC subtype distinction	-	in vitro, in silico [86]

Abbreviations: AUC, area under the curve; BC, breast cancer; circRNAs, circular RNAs; HER2, human epidermal growth factor receptor 2; HR, hazard ratio; N, normal breast tissue; ROC, receiver operating characteristic; T, breast tumor tissue; TNBC, triple-negative breast cancer.

**Table 2:** List of circulating circRNAs reported as potential biomarkers in breast cancer.

circRNA	Biological Source	Comparison	Expression in Tumor Specimens	Clinical Interest	Diagnostic Value (ROC)	Experimental Approach
hsa_circ_0009634	exosomes (serum)	metastatic T vs. localized T vs. N	up	diagnosis	-	ex vivo, in vitro, in silico [95]
hsa_circ_0020707				diagnosis	-	ex vivo, in vitro, in silico [95]
hsa_circ_0064923				diagnosis	-	ex vivo, in vitro, in silico [95]
hsa_circ_0087064				diagnosis	-	ex vivo, in vitro, in silico [95]
hsa_circ_0104852				diagnosis	-	ex vivo, in vitro, in silico [95]
circRNA_0005795		T vs. N	down	diagnosis	-	ex vivo, in vitro, in silico [96]
circRNA_0088088				diagnosis	-	ex vivo, in vitro, in silico [96]
BCexoc (hsa_circ_0002190, hsa_circ_0007177, hsa_circ_0000642, hsa_circ_0001439, hsa_circ_0001417, hsa_circ_0005552, hsa_circ_0001073, hsa_circ_0000267, hsa_circ_0006404)	exosomes (plasma)			early diagnosis	AUC = 0.83	ex vivo, in silico [97]
circCNOT2	plasma	T vs. N	up	prognosis, prediction of therapy response	-	ex vivo, in vitro, in silico [98]
hsa_circ_0001785				early diagnosis, prediction of therapy response	AUC = 0.784, Sensitivity = 0.764, Specificity = 0.699	ex vivo, in silico [99]
hsa_circ_0008673				diagnosis, prognosis	AUC = 0.833, Specificity = 97.10%, Sensitivity = 55%, HR = 1.742, *p* = 0.047	ex vivo, in vitro, in silico [100,101]
hsa_circ_0017536				diagnosis	AUC = 615, *p* < 0.05	ex vivo, in vitro, in silico [102]
hsa_circ_0017650				diagnosis	AUC = 0.758, *p* < 0.05	ex vivo, in vitro, in silico [102]
hsa_circ_0069094				diagnosis	AUC = 0.681, *p* < 0.05	ex vivo, in vitro, in silico [102]
hsa_circ_0079876				diagnosis	AUC = 0.623, *p* < 0.05	ex vivo, in vitro, in silico [102]
hsa_circ_0104824				diagnosis	AUC = 0.849, *p* = 0.0001, Sensitivity = 71.1%, Specificity = 75.5%	ex vivo, in silico [103]
circCDYL (hsa_circ_0008285)	serum			diagnosis, prognosis, prediction of therapy response	HR = 3.748, *p* = 0.002	ex vivo, in vitro, in vivo, in silico [104]
circKIF4A (circ_0007255)				diagnosis, prognosis	AUC = 0.77	ex vivo, in vitro, in vivo, in silico [105]

Abbreviations: AUC, area under the curve; circRNAs, circular RNAs; HR, hazard ratio; N, normal samples; T, tumor samples; ROC, receiver operating characteristic.

**Table 3:** List of circRNAs involved in resistance to BC therapies.

circRNA Name	Circbase ID	Resistance/*Sensitivity* to:	Experimental Approach
cirCDR1as	hsa_circ_0001946	*5-fluorouracil; cisplatin*	ex vivo, in vitro, in vivo, in silico [123,124]
circSMARCA5	hsa_circ_0001445	*cisplatin*	ex vivo, in vitro, in vivo, in silico [125]
circKDM4C	hsa_circ_0001839	*doxorubicin*	ex vivo, in vitro, in vivo, in silico [126]
circLARP4	-	*doxorubicin*	ex vivo, in vitro [67]
circUBE2D2	hsa_circ_0005728	doxorubicin	ex vivo, in silico [79]
-	hsa_circ-0006528	doxorubicin	in vitro, in silico [127]
-	hsa_circ_0092276	doxorubicin	in vitro, in vivo, in silico [128]
circBMPR2	hsa_circ_0003218	*tamoxifen*	ex vivo, in vitro, in silico [129]
-	hsa_circ_0025202	tamoxifen	ex vivo, in vitro, in vivo, in silico [130]
circABCB1	-	taxane (docetaxel)	in vitro, in silico [131]
circEPHA3.1	-	taxane (docetaxel)	in vitro, in silico [131]
circEPHA3.2	-	taxane (docetaxel)	in vitro, in silico [131]
circAMOTL1	-	taxane (paclitaxel)	in vitro, in silico [132]
circGFRA1	hsa_circ_0005239	taxane (paclitaxel)	in vitro, in vivo [74]
circRNF111	hsa_circ_0001982	taxane (paclitaxel)	ex vivo, in vitro, in vivo [133]
circABCB10	-	taxane (paclitaxel); radiations	ex vivo, in vitro, in vivo, in silico [134,135]
circHER2	hsa_circ_0007766	*pertuzumab*	ex vivo, in vitro, in vivo, in silico [136]

Abbreviations: circRNA, circular RNA.
